# Supplementary material for: T cell microvilli constitute immunological synaptosomes that carry messages to antigen-presenting cells
Source: Nat Commun. 2018 Sep 7;9:3630. doi: 10.1038/s41467-018-06090-8 (PMC6128830; doi:10.1038/s41467-018-06090-8)
Supplement: Supplementary file 1 — Supplementary Information [file 41467_2018_6090_MOESM1_ESM.pdf]

Supplementary Information

T-cell microvilli constitute immunological synaptosomes that carry messages to antigen-presenting cells

Kim et al.

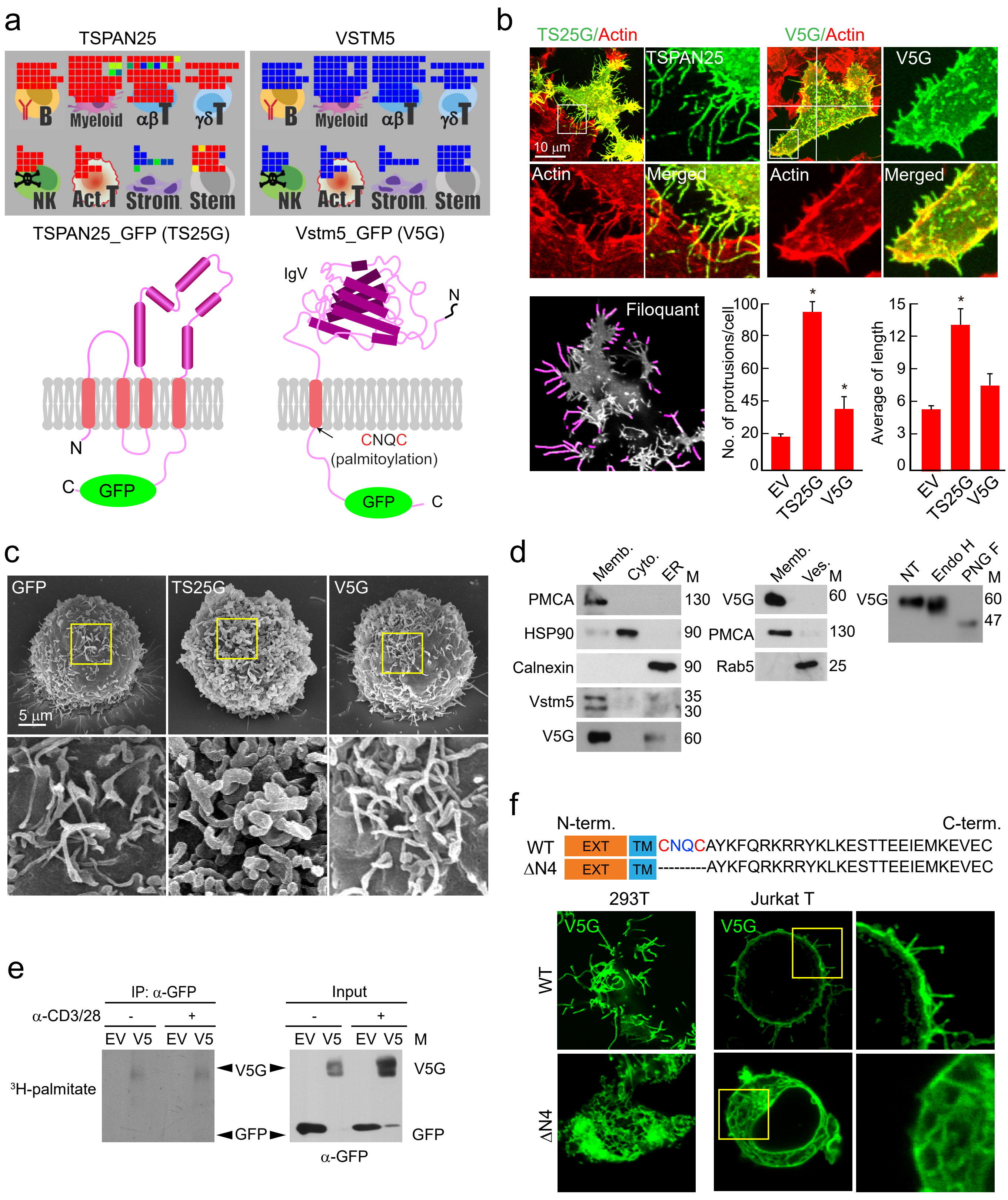
a
TSPAN25
VSTM5
B
Myeloid
αβT
γδT
NK
Act.T
Strom.
Stem
TSPAN25_GFP (TS25G)
Vstm5_GFP (V5G)
IgV
N
C
GFP
CNQC
(palmitoylation)
b
TS25G/Actin
V5G/Actin
TSPAN25
V5G
10 μm
Actin
Merged
Filoquant
No. of protrusions/cell
Average of length
EV
TS25G
V5G
c
GFP
TS25G
V5G
5 μm
d
Memb.
Cyto.
ER
M
PMCA
HSP90
Calnexin
Vstm5
V5G
Ves.
Rab5
NT
Endo H
PNG F
e
IP: α-GFP
Input
α-CD3/28
EV V5 EV V5
³H-palmitate
V5G
GFP
α-GFP
f
N-term.
C-term.
WT
ΔN4
EXT
TM
CNQCAYKFQRKRRYKLKESTTEEIEMKEVEC
---------AYKFQRKRRYKLKESTTEEIEMKEVEC
293T
Jurkat T

Supplementary Fig. 1. Localization of TSPAN25 and Vstm5 in HEK293T cells and T cells (**a**) Expression of TSPAN25 and Vstm5 in immune cell subsets from the Immunological Genome Project. Red and blue colours indicate high and low expressions, respectively (*top*). Schematic representation of TSPAN25_GFP (TS25G) and Vstm5_GFP (V5G) on the cell membrane (*bottom*). (**b**) Morphological assessment of HEK293T cells after overexpression of TS25G or V5G. Quantification of the number of microvilli and their average length by FiloQuant. *$P < 0.05$ *versus* EV. (**c**) Jurkat T cells expressing TS25G or V5G were plated on PLL-coated coverglass and observed under SEM. Boxed regions are shown enlarged at the right of the panel. (**d**) Subcellular fractionation of Vstm5 or V5G in Jurkat T cells. Proteins from membrane, cytosolic, ER, and vesicle fractions were immunoblotted with the antibodies as indicated. PMCA (calcium pump pan PMCA ATPase), HSP90, Calnexin, and Rab5 were positive markers for membrane, cytosol, ER, and vesicle, respectively. T-cell lysates containing V5G were digested with Endo H or PNGase F and were immunoblotted with anti-GFP antibody. (**e**) Autoradiograph showing [^{3}H]-palmitic acid incorporation in V5G expressed in Jurkat T cells. (**f**) Schematic representation of the palmitoylated sequence of Vstm5 and subcellular localization of V5GΔN4 mutant in 293T or Jurkat T cells.

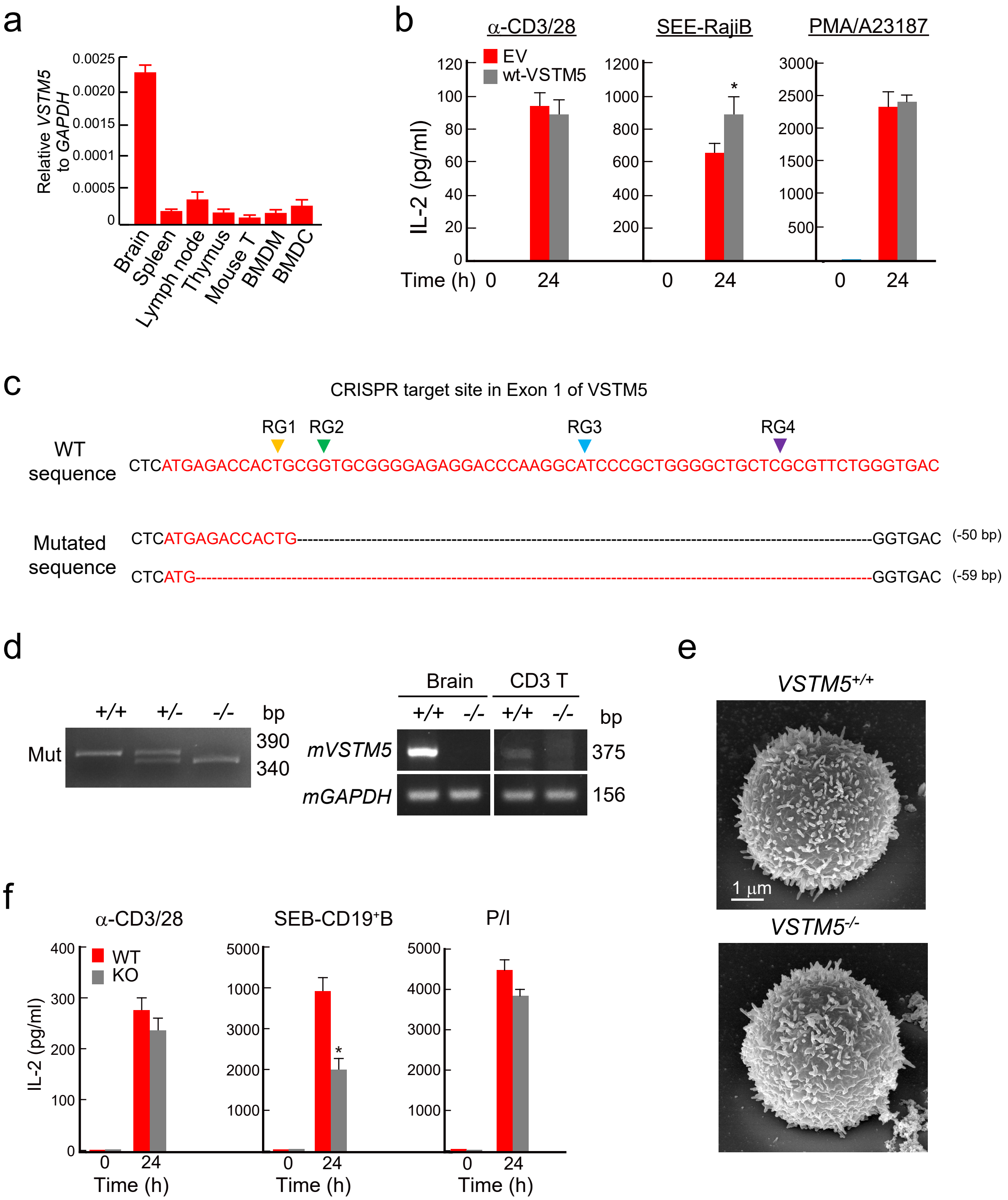
a
Relative VSTM5 to GAPDH
0.0025
0.0020
0.0015
0.0001
0.0005
0
Brain
Spleen
Lymph node
Thymus
Mouse T
BMDM
BMDC
b
α-CD3/28
SEE-RajiB
PMA/A23187
EV
wt-VSTM5
IL-2 (pg/ml)
120
100
80
60
40
20
0
1200
1000
800
600
400
200
3000
2500
2000
1500
1000
500
*
Time (h) 0 24
0 24
0 24
c
CRISPR target site in Exon 1 of VSTM5
RG1 RG2 RG3 RG4
WT sequence
CTCATGAGACCACTGCGGTGCGGGGAGAGGACCCAAGGCATCCCGCTGGGGCTGCTCGCGTTCTGGGTGAC
Mutated sequence
CTCATGAGACCACTG------GGTGAC (-50 bp)
CTCATG------GGTGAC (-59 bp)
d
+/+ +/- -/- bp
Mut
390
340
Brain
CD3 T
+/+ -/- +/+ -/- bp
mVSTM5
375
mGAPDH
156
e
VSTM5+/+
1 µm
VSTM5-/-
f
α-CD3/28
SEB-CD19+B
P/I
WT
KO
IL-2 (pg/ml)
400
300
200
100
0
5000
1000
3000
2000
1000
*
5000
4000
3000
2000
1000
0 24
Time (h)
0 24
Time (h)
0 24
Time (h)

Supplementary Fig. 2. Functions of Vstm5 in T cells (**a**) Comparison of *Vstm5* expression in various tissues and immune cells by RT-qPCR. Relative target gene expression levels were normalized to those of *Gapdh*. The data represent the mean of three experiments ± SEM. (**b**) Effect of V5G overexpression in Jurkat T cells to produce IL-2 in response to various stimuli including anti-CD3/28, incubation with SEE-loaded Raji B cells, and PMA (200 nM)/ionomycin (1 μM). The data represent the mean of three experiments ± SEM. $^{*}P < 0.05$ *versus* EV. (**c**) Schematic diagram of the construction of $Vstm5^{-/-}$ mice using the CRISPR/Cas9 method. Mutation was detection by T-cloning and Sanger sequencing. The wild-type (WT) sequence is shown at the top of the targeting sequence. RG1, 2, 3, and 4 represent single guide RNA (sgRNA) targeting sites. (**d**) Representative conventional PCR for genotype and *Vstm5* expression in brain and $CD3^{+}$ T cells from the wild-type and $Vstm5^{-/-}$ mice. (**e**) SEM images of wild-type and $Vstm5^{-/-}$ mouse T cells on PLL. (**f**) Effect of deletion of Vstm5 in mouse T cells to produce IL-2 in response to various stimuli including anti-CD3/28, incubation with SEB-loaded mouse B cells, and PMA (200 nM)/ionomycin (1 μM). The data represent the mean of three experiments ± SEM. $^{*}P < 0.05$ *versus* EV.

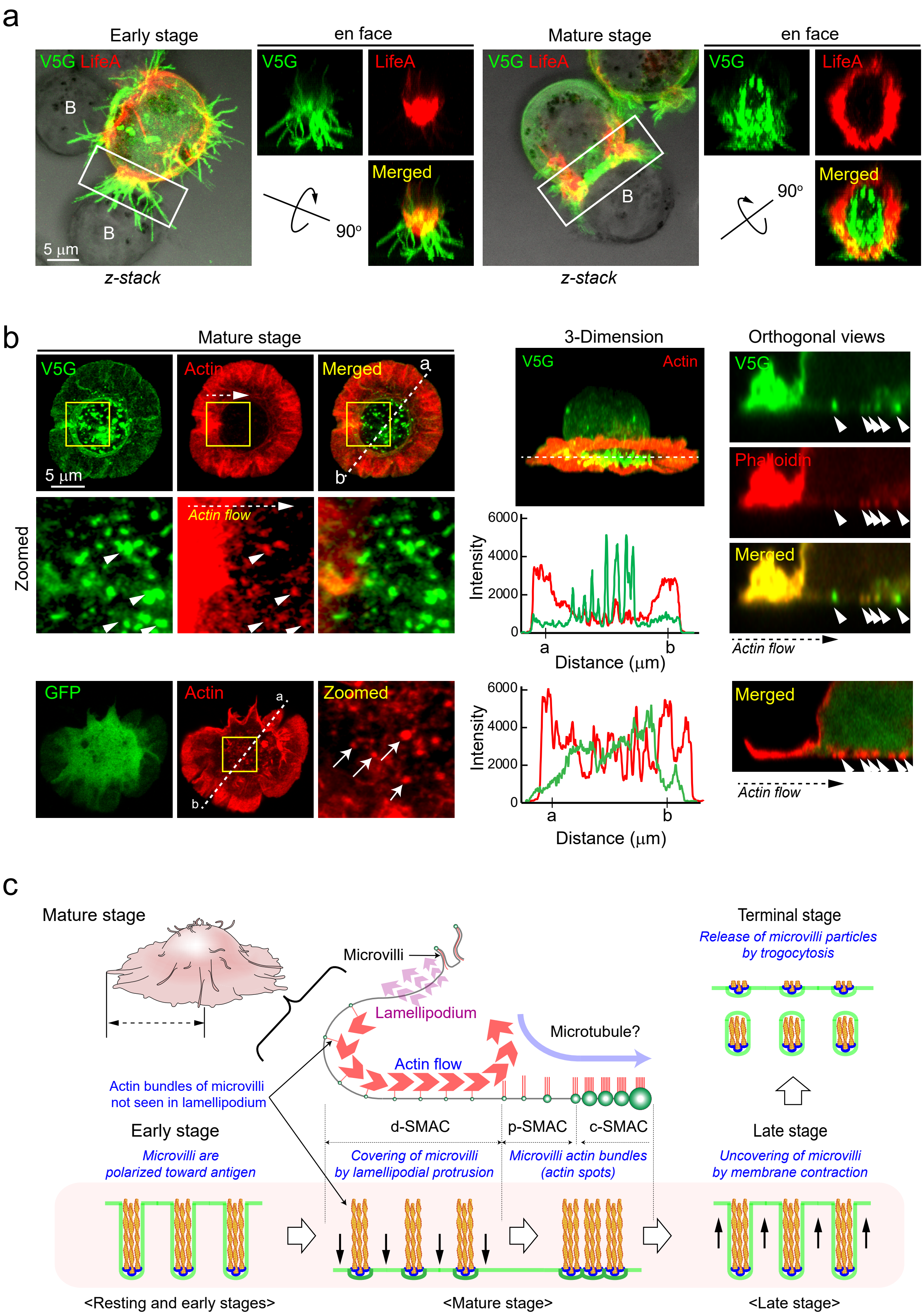
a
Early stage
en face
Mature stage
en face
V5G LifeA
V5G
LifeA
Merged
B
B
5 µm
90°
z-stack
b
Mature stage
3-Dimension
Orthogonal views
V5G
Actin
Merged
a
b
5 µm
Zoomed
Actin flow
Phalloidin
Intensity
Distance (µm)
GFP
Zoomed
c
Mature stage
Microvilli
Lamellipodium
Microtubule?
Actin flow
Actin bundles of microvilli not seen in lamellipodium
Terminal stage
Release of microvilli particles by trogocytosis
Early stage
Microvilli are polarized toward antigen
d-SMAC
p-SMAC
c-SMAC
Covering of microvilli by lamellipodial protrusion
Microvilli actin bundles (actin spots)
Late stage
Uncovering of microvilli by membrane contraction
<Resting and early stages>
<Mature stage>
<Late stage>

Supplementary Fig. 3. **Localization of V5G with F-actin spots during IS formation** (**a**) Polarization of V5G$^+$ microvilli toward the T cell–APC interface. Jurkat T cells co-expressing V5G and LifeAct_mRFP (red color) were conjugated with SEE-Raji B cells for 5 (early) or 30 min (mature). 3D reconstruction of the each stage and the projected en face are shown. (**b**) GFP$^+$ or V5G$^+$ Jurkat T cells were placed on an anti-CD3 coverglass for 5 min, fixed, and stained with TRITC-phalloidin. The fluorescence intensity profiles from a to b were analysed. Yellow arrows indicate co-localization of V5G and F-actin along with the actin flow. See also Supplementary Movie 2. (**c**) Schematic diagram presenting a potential mechanism of how microvilli that disappear at the dSMAC move toward the cSMAC with TCR clusters during IS formation, and reappear after IS deformation.

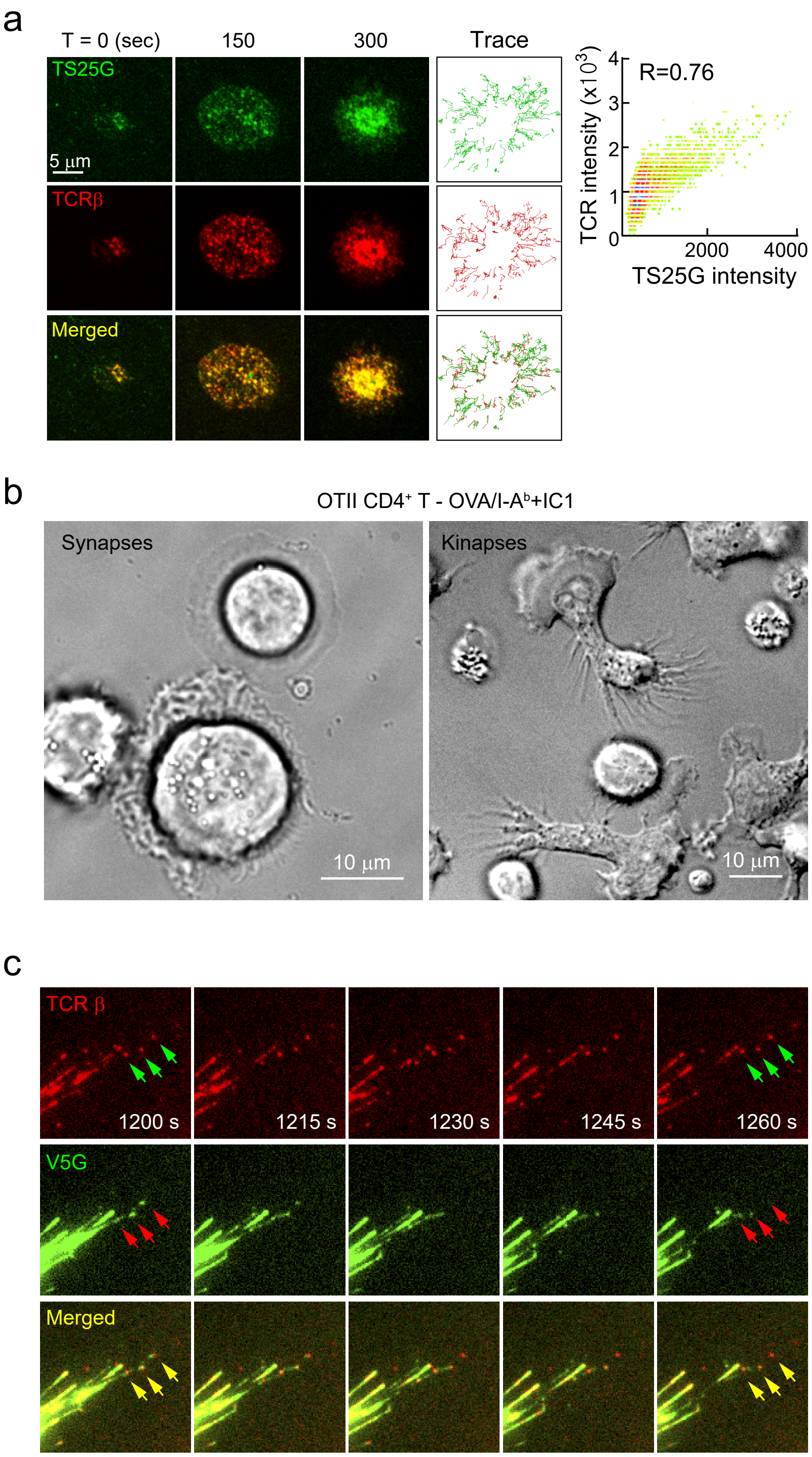
a
T = 0 (sec)
150
300
Trace
TS25G
5 μm
TCRβ
Merged
R=0.76
TCR intensity (x10^3)
TS25G intensity
b
OTII CD4+ T - OVA/I-Ab+IC1
Synapses
Kinapses
10 μm
c
TCR β
1200 s
1215 s
1230 s
1245 s
1260 s
V5G
Merged

Supplementary Fig. 4. Co-localization of TS25G with TCR clusters and centripetal movements into the cSMAC (**a**) TS25G$^+$ OTII CD4$^+$ T cells stained with anti-TCRβ (H57Fab-594) were examined on a planar bilayer presenting OVA peptide/I-A^b and ICAM-1. Images were selected at 150 s and 300 s post-recording. Representative trajectories of individual TS25G and TCR clusters are shown in the right panels. The co-localization was estimated by the Pearson's correlation coefficient (R). (**b**) Live imaging of OTII CD4$^+$ T cells on lipid bilayer presenting OVA peptide/I-A^b and ICAM-1 during synapses and kinapses under the phase-contrast microscopy. (**c**) Still frames of TCRβ$^+$/V5G$^+$-microvillus particle release from T cells in kinapses (corresponding images of Fig. 3d and Supplementary Movie 4. Red: TCR, Green: V5G).

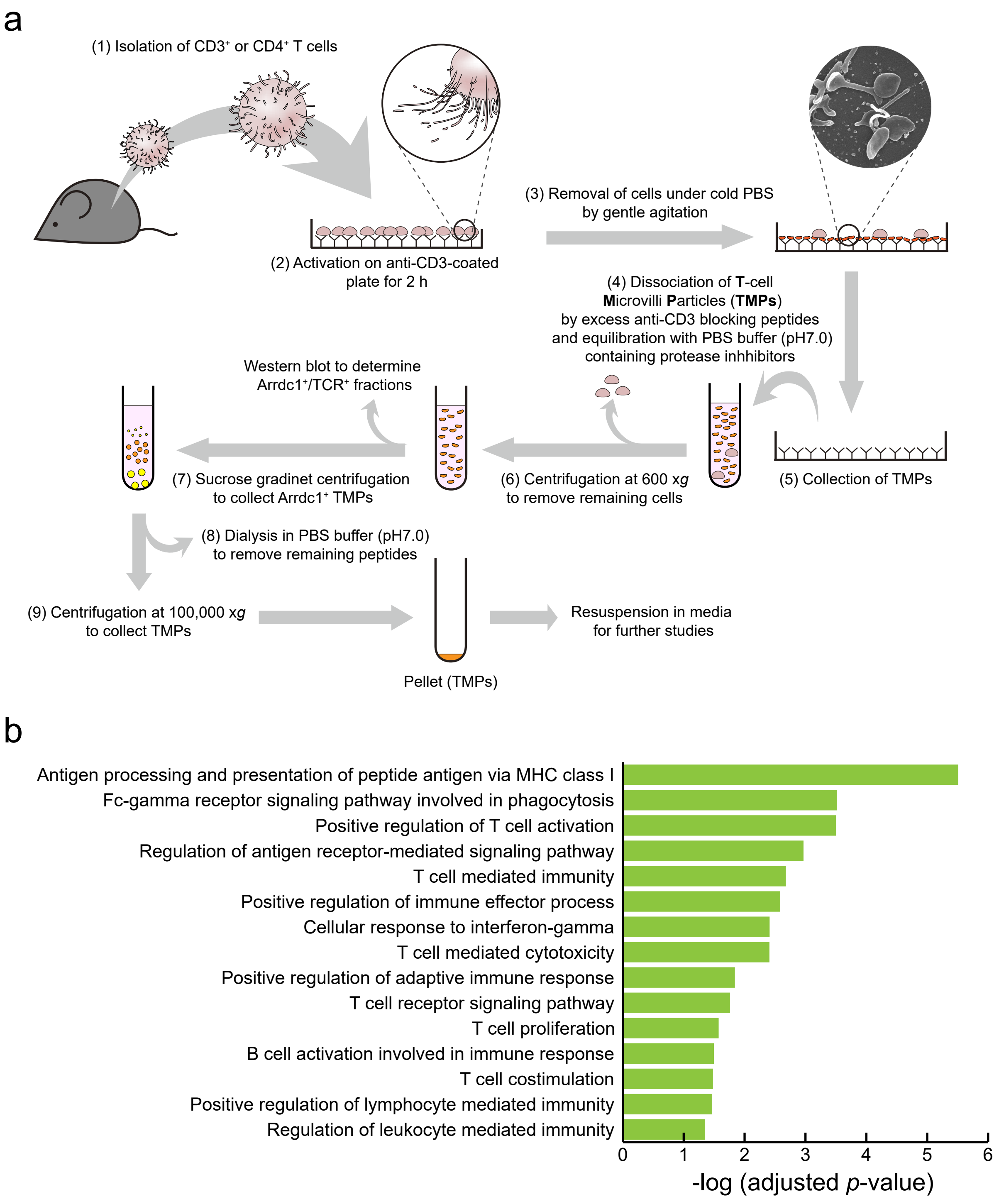

Supplementary Fig. 5. **Purification and composition of TMPs** (**a**) Schematic procedures for TMP purification from mouse or human T cells as described in the Methods. (**b**) Representative immune system processes of the TMP proteome. ClueGO determined the distribution of the protein list of TMPs for the various GO immune system process terms [1,2]. The p-value was calculated using the right-sided hypergeometric test and Benjamini-Hochberg correction for multiple testing. The p-values are expressed as the negative logarithm (base 10).

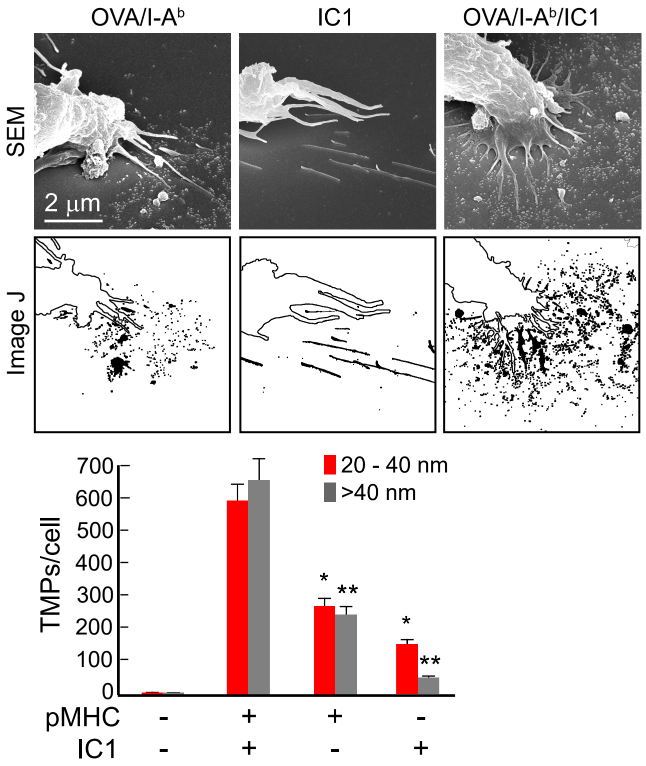

Supplementary Fig. 6. **TMP generation requires both TCR and adhesion signaling** OTII CD4+ T cells were placed on lipid bilayer (OVA peptide/I-A^b only, ICAM-1 only, and OVA peptide/I-A^b and ICAM-1). After 1 h, TMPs were quantitated. Processed images at the bottom of the panels represent the TMPs after image processing. Potential TMPs were outlined with a black line, with the inside filled with black color, and then counted by Image J software. Data represent the mean of three experiments ± SEM. *$P < 0.05$ *versus* pMHC/IC1 (20–40 nm), **$P < 0.01$ *versus* pMHC/IC1 (>40 nm).

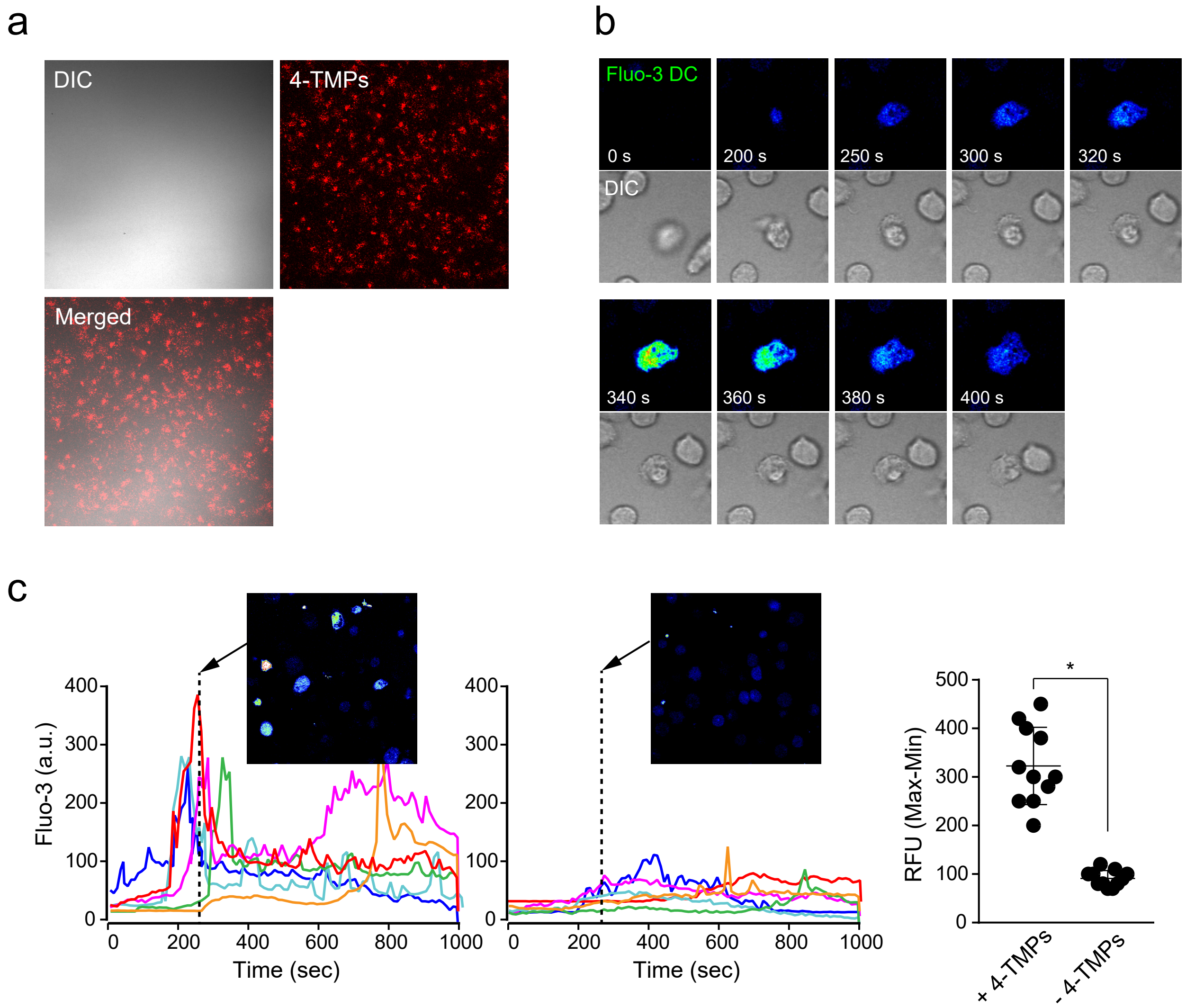

Supplementary Fig. 7. 4-TMPs activate calcium responses in DCs independent of TCR engagement (**a**) Removal of all T cells on lipid bilayers after release of TMPs. OTII $CD4^+$ T cells were stained with anti-TCRβ (H57Fab-Alexa594) and seeded on a lipid bilayer presenting OVA peptide/I-A^b and ICAM-1 for 2 h. Cells were then removed by washing with cold HBS/HAS. The images were observed by confocal microscopy. (**b**) Still frames from time-lapse imaging of Fluo-3-loaded DCs placed on 4-TMP-deposited lipid bilayer (corresponding images of Fig. 8a). Note, Fluo-3 and DIC images are separated. (**c**) Quantification of Fluo-3 fluorescence intensity in DCs placed on '+TMPs' or '-TMPs'. *P < 0.01 *versus* -TMPs.

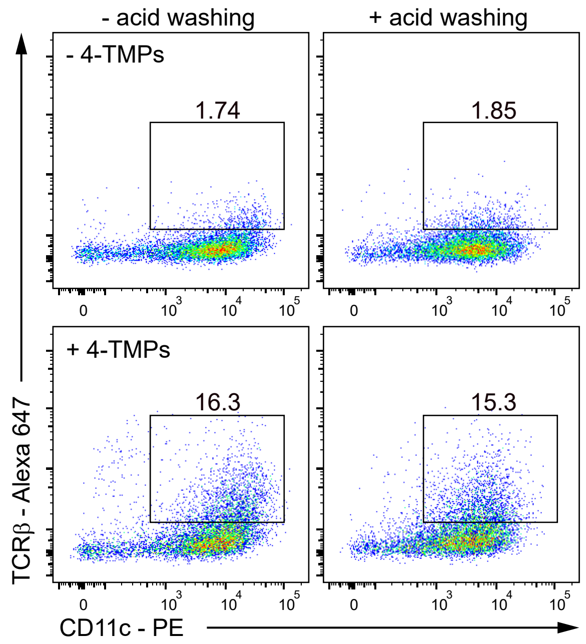

Supplementary Fig. 8. Comparison before and after acid washing DCs (5×10^6) were treated with PBS or 4-TMPs (5×10^7) for 2 h, and then the cells were washed with acid buffer and stained for the indicated surface markers.

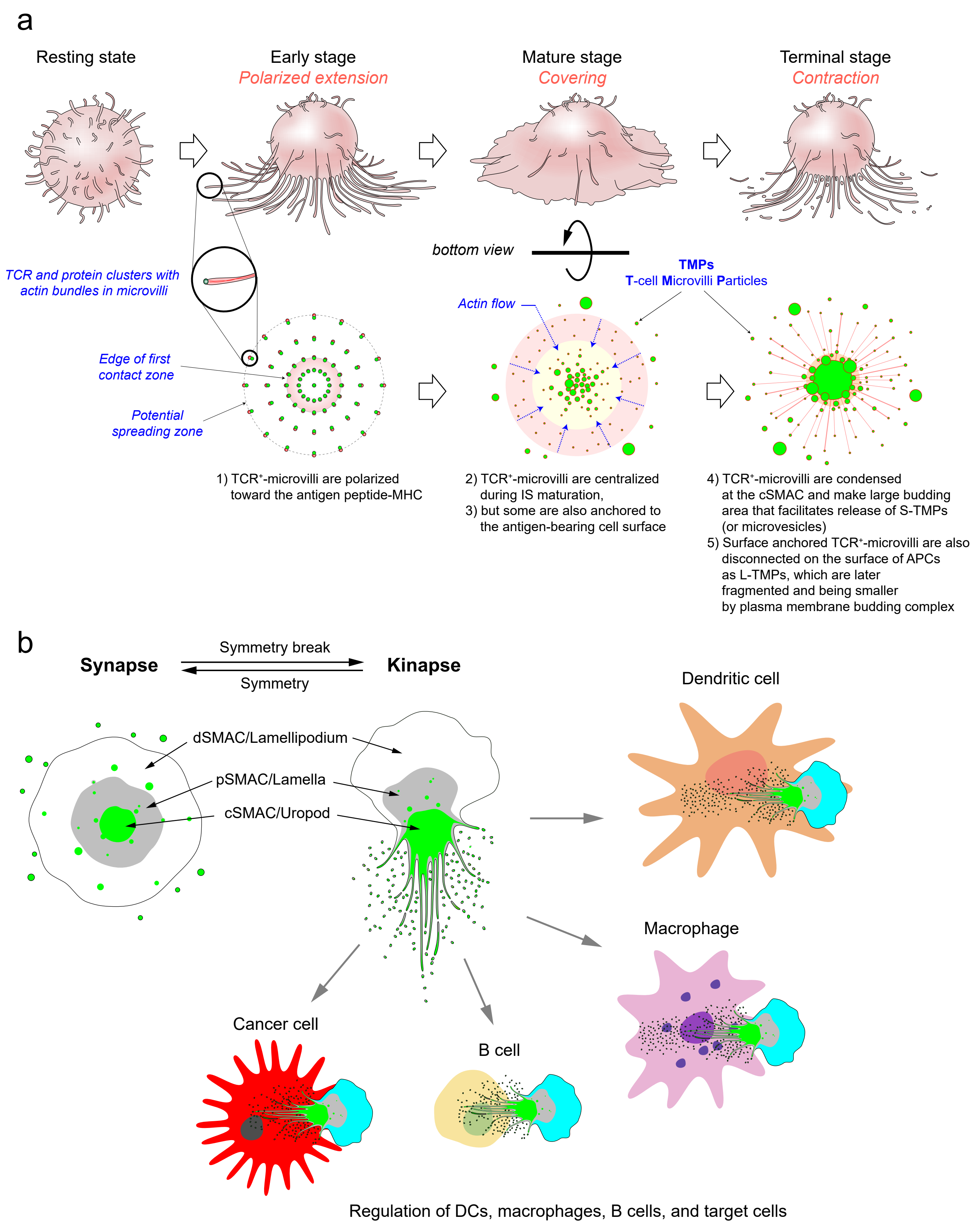
a
Resting state
Early stage
Polarized extension
Mature stage
Covering
Terminal stage
Contraction
bottom view
TCR and protein clusters with actin bundles in microvilli
Edge of first contact zone
Potential spreading zone
Actin flow
TMPs
T-cell Microvilli Particles
1) TCR+-microvilli are polarized toward the antigen peptide-MHC
2) TCR+-microvilli are centralized during IS maturation,
3) but some are also anchored to the antigen-bearing cell surface
4) TCR+-microvilli are condensed at the cSMAC and make large budding area that facilitates release of S-TMPs (or microvesicles)
5) Surface anchored TCR+-microvilli are also disconnected on the surface of APCs as L-TMPs, which are later fragmented and being smaller by plasma membrane budding complex
b
Synapse
Symmetry break
Symmetry
Kinapse
dSMAC/Lamellipodium
pSMAC/Lamella
cSMAC/Uropod
Dendritic cell
Macrophage
Cancer cell
B cell
Regulation of DCs, macrophages, B cells, and target cells

Supplementary Fig. 9. Schematic model showing TMP generation from activated T cells
(**a**) During IS formation, 1) TCR-enriched microvilli on T cells are polarized toward the antigen peptide-MHC on APCs; 2) TCR^+-microvilli are centralized during IS maturation; but 3) some are also anchored to the antigen-bearing cell surface; 4) TCR^+-microvilli are condensed at the cSMAC and make large budding area that facilitates release of S-TMPs (or microvesicles); and 5) Surface anchored TCR^+-microvilli are also disconnected on the surface of APCs as L-TMPs, which are later fragmented and being smaller by plasma membrane budding complex such as Arrdc1, TSG101, and Vps4. (**b**) During kinaptic phase, T cells release abundant TMPs that may regulate further T-cell-mediated immune responses.

Fig. 6c

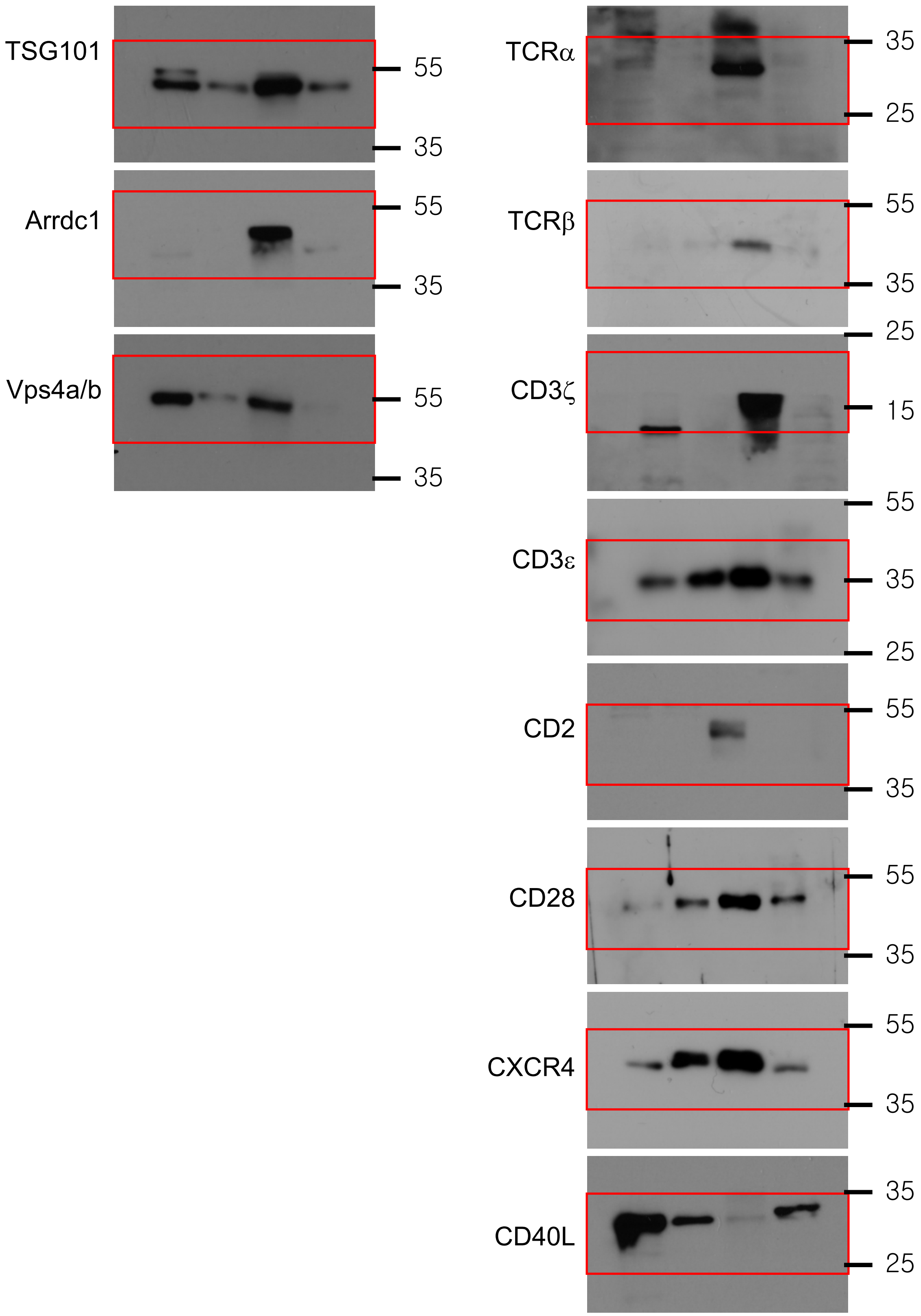

Supplementary Fig. 10. Uncropped Western blots corresponding to different panels. Boxes with dashed lines in red color indicate the areas cropped and used in main figures.

Fig. 6c

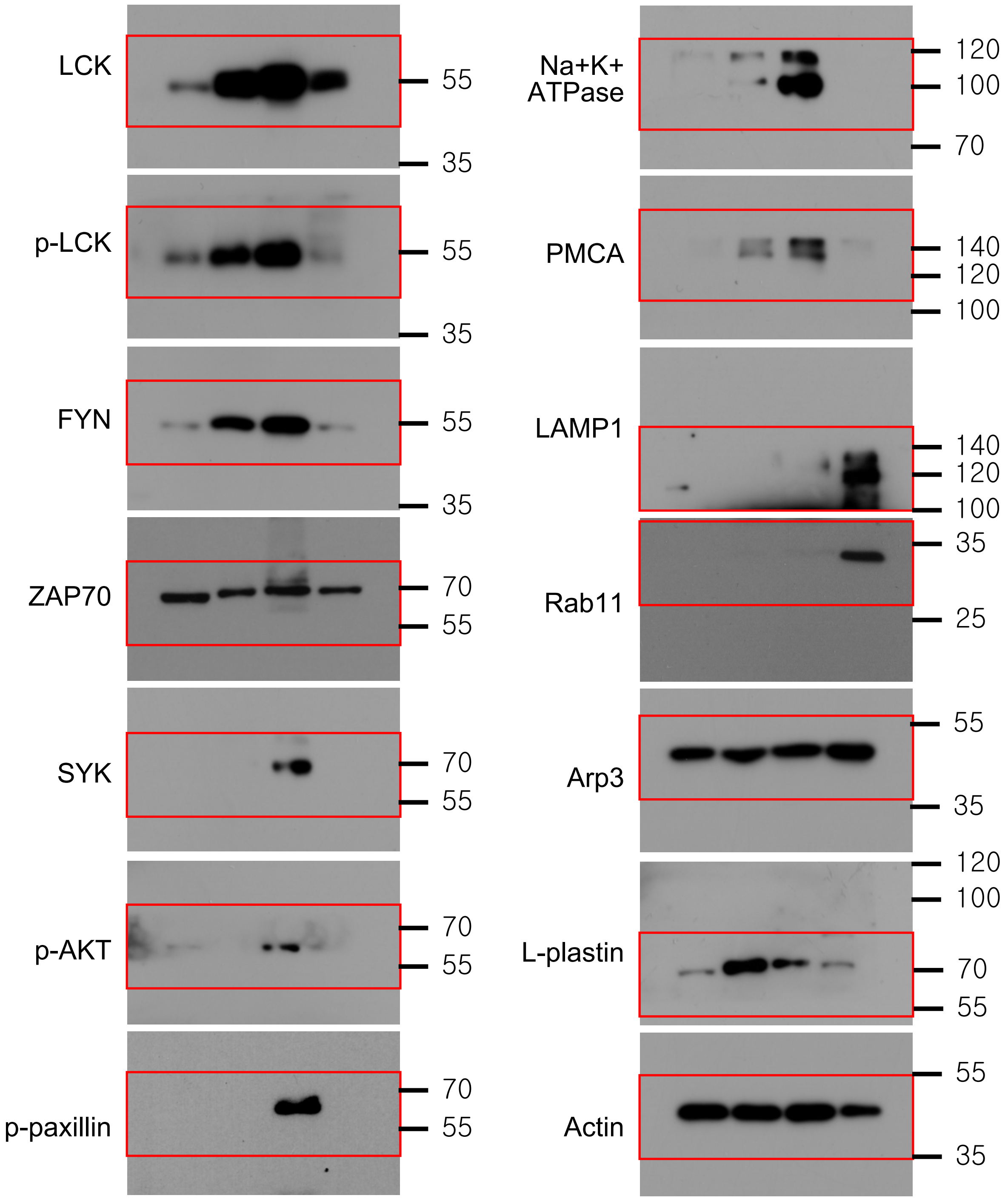

Supplementary Fig. 11. Uncropped Western blots corresponding to different panels.
Boxes with dashed lines in red color indicate the areas cropped and used in main figures.

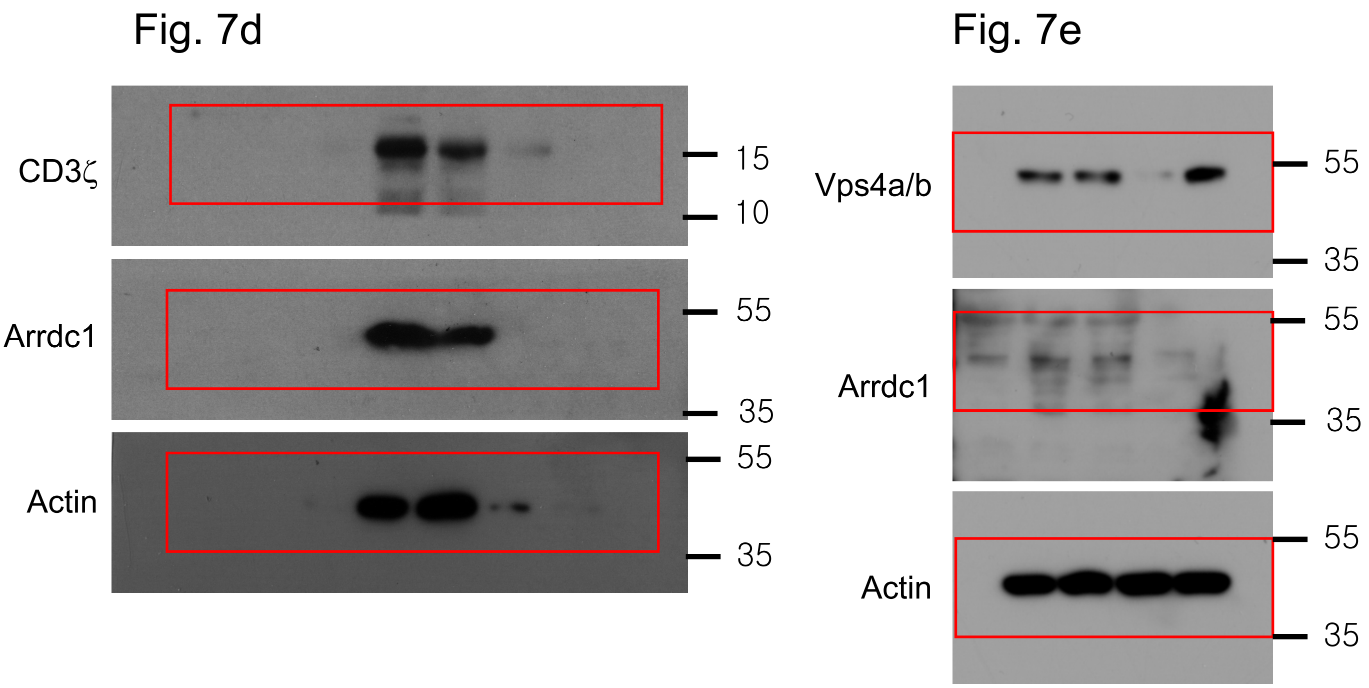

Supplementary Fig. 12. Uncropped Western blots corresponding to different panels. Boxes with dashed lines in red color indicate the areas cropped and used in main figures.

REFERENCES

1. Bindea, G. *et al.* ClueGO: A Cytoscape plug-in to decipher functionally grouped gene ontology and pathway annotation networks. *Bioinformatics* **25,** 1091–1093 (2009).

2. Shannon, P. *et al.* Cytoscape: a software environment for integrated models of biomolecular interaction networks. *Genome Research* 2498–2504 (2003). doi:10.1101/gr.1239303.metabolite
