# Supplementary material for: T cell microvilli constitute immunological synaptosomes that carry messages to antigen-presenting cells
Source: Nat Commun. 2018 Sep 7;9:3630. doi: 10.1038/s41467-018-06090-8 (PMC6128830; doi:10.1038/s41467-018-06090-8)
Supplement: Supplementary file 3 — Description of Additional Supplementary Files [file 41467_2018_6090_MOESM3_ESM.pdf]

[Legends for Supplementary data and movies]

Supplementary data. The proteins that were commonly detected in three independent NanoLC-ESI-MSMS in the total, membrane, TMP, and vesicle groups were selected and are listed. [a]Proteins were ranked in order of peptide spectral matches. Accession numbers are from SwissProt.

Supplementary Movie 1. **Separation of V5G^{+}-microvillus particles from Jurkat T cells on anti-CD3-coated coverglass.** V5G^{+}- Jurkat T cells were placed on an anti-CD3-coated coverglass, then the generation of V5G^{+}-microvilli particles was observed by TIRFM (IX-81; Olympus). Time-lapse images were taken at 12-s intervals for 30 min. This video corresponds to Fig. 2f.

Supplementary Movie 2. Three-dimensional localization of V5G (green) and F-actin (red) during IS formation. At the mature stage on an anti-CD3 antibody-coated surface, V5G^{+} Jurkat T cells were fixed and stained with TRITC-phalloidin. Z-stack images were taken by confocal microscope. 3D image reconstruction was performed using Imaris software. *Note,* V5G is colocalized with F-actin spots in the inner circle of IS. This video corresponds to Supplementary Fig. 3b.

Supplementary Movie 3. Centripetal movements of V5G together with TCR during IS formation. V5G^{+} OTII CD4^{+} T blasts stained with TCRβ (H57Fab-594) were placed on a planar lipid bilayer presenting OVA323-339- I-A^{b}/ICAM-1 and were immediately imaged for 30 min every 15 s under TIRFM. Relative time scales starting from the moment of cell spreading are labeled on the movie. This video corresponds to Fig. 3a.

Supplementary Movie 4. **Separation of TCRβ^{+}/V5G^{+}-microvillus particles from T cells during immunological kinapses.** V5G^{+} OTII CD4^{+} T blasts were placed on a planar lipid bilayer presenting OVA323-339- I-A^{b}/ICAM-1. Cells were imaged at least for 2 h under TIRFM, and then moving T cells in the kinapses were selected for further image processing. This video corresponds to Fig. 3d.

Supplementary Movie 5. CD4^{+} T cells leave V5G^{+}-microvillus particles during T cell interaction with DCs. V5G^{+} CD4^{+} T blasts from wild-type mice were incubated with SEB-loaded, CMRA-stained DCs for 10 min. Time-lapse images of L-TMP generation from the T cells were taken at every 24 s for 60 min under confocal microscopy (LSM 880; Zeiss). Multiple contact sites are indicated by white arrows and the L-TMPs are indicated by yellow arrows. This video corresponds to Fig. 4b (upper panel).

Supplementary Movie 6. Microparticles from DCs are transferred to T cells via T-cell microvilli. V5G^{+}CD4^{+} T blasts from wild-type mice were incubated with SEB-loaded, CMRA-stained DCs for 10 min. Microparticles (red arrow) from DCs were transferred to microvilli on T cells. Time-lapse images were taken at every 30 s for 60 min under confocal microscopy (LSM 880; Zeiss). Multiple contact sites are indicated by white arrows and particles from DCs are indicated by red arrows. This video corresponds to Fig. 4b (bottom panel).
